# Adhesive F-actin Waves: A Novel Integrin-Mediated Adhesion Complex Coupled to Ventral Actin Polymerization

**DOI:** 10.1371/journal.pone.0026631

**Published:** 2011-11-01

**Authors:** Lindsay B. Case, Clare M. Waterman

**Affiliations:** 1 Department of Cell and Developmental Biology, University of North Carolina School of Medicine, Chapel Hill, North Carolina, United States of America; 2 Cell Biology and Physiology Center, National Heart, Lung, and Blood Institute, National Institutes of Health, Bethesda, Maryland, United States of America; University of Muenster, Germany

## Abstract

At the leading lamellipodium of migrating cells, protrusion of an Arp2/3-nucleated actin network is coupled to formation of integrin-based adhesions, suggesting that Arp2/3-mediated actin polymerization and integrin-dependent adhesion may be mechanistically linked. Arp2/3 also mediates actin polymerization in structures distinct from the lamellipodium, in “ventral F-actin waves” that propagate as spots and wavefronts along the ventral plasma membrane. Here we show that integrins engage the extracellular matrix downstream of ventral F-actin waves in several mammalian cell lines as well as in primary mouse embryonic fibroblasts. These “adhesive F-actin waves” require a cycle of integrin engagement and disengagement to the extracellular matrix for their formation and propagation, and exhibit morphometry and a hierarchical assembly and disassembly mechanism distinct from other integrin-containing structures. After Arp2/3-mediated actin polymerization, zyxin and VASP are co-recruited to adhesive F-actin waves, followed by paxillin and vinculin, and finally talin and integrin. Adhesive F-actin waves thus represent a previously uncharacterized integrin-based adhesion complex associated with Arp2/3-mediated actin polymerization.

## Introduction

Cell migration is a coordinated event involving protrusion, adhesion to the extracellular matrix (ECM), myosin II-driven contraction of the cell body, and adhesion disassembly at the cell rear. In the lamellipodium, protrusion of an Arp2/3-nucleated actin network is coupled to formation of integrin-based adhesions [1]. Arp2/3-mediated actin polymerization and integrin-dependent adhesion may be mechanistically linked, as the rate of adhesion assembly is directly correlated with the rate of lamellipodial protrusion [1], and the focal adhesion proteins vinculin and focal adhesion kinase (FAK) have been shown to interact with Arp2/3 [2]–[3]. While the Arp2/3-nucleated dendritic actin network is a defining characteristic of the lamellipodium, Arp2/3-dependent actin polymerization is not limited to this structure. Arp2/3-dependent actin polymerization is important for the formation of the immunological synapse, endocytosis and vesicle fusion, membrane ruffling, and ventral F-actin waves [4].

Ventral F-actin waves have been characterized in neutrophils, fibroblasts, and Dictyostelia [5]–[7]. In spite of their conservation across eukaryotic cells, the function of ventral F-actin waves is not well understood. In neutrophils, F-actin waves are induced by chemoattractant and are proposed to mediate cell migration [5], while in Dictyostelium, they are thought to be involved in phagocytosis [8]. Ventral F-actin waves occur when actin spontaneously nucleates and polymerizes on the ventral, substrate-attached surface of cells, independently of the cell edge [7], [9]. This polymerizing actin can form discrete spots, moving spots, or propagate in semicircular wave patterns [10].

Several studies have begun to characterize the mechanism of ventral F-actin wave formation and propagation. In Dictyostelia, myosin II does not localize to ventral F-actin waves and the formation and motion of ventral F-actin waves occurs in myosin II null cells [11]. However, their sensitivity to actin polymerization inhibitors and fluorescence recovery after photobleaching (FRAP) experiments indicate that ventral F-actin waves propagate by actin polymerization and treadmilling [5], [11]. Localization studies have shown that ventral F-actin waves contain Arp2/3 and its activator, the WAVE complex, suggesting their involvement in stimulating actin treadmilling [5], [7]. Actin assembly by Arp2/3 in ventral F-actin waves may be mediated by a PI3K/Rac1 signaling cascade, since they are sensitive to the PI3K inhibitor LY294002, [8], [12] and active Rac1 forms propagating wave patterns similar to ventral F-actin waves [5]. Together, these data suggest that PI3K and Rac1 promote WAVE- and Arp2/3-dependent actin treadmilling to form ventral F-actin waves and drive their propagation.

In spite of the knowledge on the mechanism of actin polymerization in ventral F-actin waves, whether they are associated with integrin-based attachment to the ECM is unknown. In this study we show that integrins engage the extracellular matrix (ECM) downstream of ventral F-actin waves. These “adhesive F-actin waves” require a cycle of integrin engagement and disengagement to the ECM for their formation and propagation. We show that the morphometry and hierarchical assembly and disassembly pathway of adhesive F-actin waves is distinct from previously characterized integrin-based adhesion structures including podosomes and focal adhesions (FAs). Adhesive F-actin waves thus represent a previously uncharacterized integrin-based adhesion complex associated with Arp2/3-mediated actin polymerization.

## Results

### Ventral F-actin waves are followed by integrin waves

**Figure 1:**
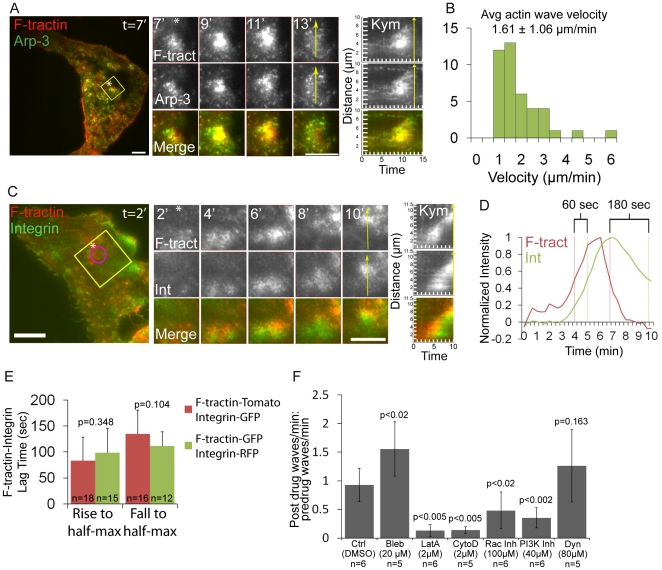
Arp2/3-containing ventral F-actin waves are followed by integrin waves. (A) LEFT: Total internal reflection fluorescence microscopy (TIRFM) image of a U2OS cell expressing F-tractin-tdtomato to label actin filaments (red) and Arp3-GFP (green). Scale bar = 10 µm. The white asterisk is to aid in orientation of the region. CENTER: Images from a time-lapse series of the region highlighted by a yellow box, time in min shown. Scale bar = 10 µm. The white asterisk is to aid in orientation of the region. RIGHT: Kymograph along the trajectory of ventral F-actin wave propagation (highlighted with a yellow arrow at t = 13 min) demonstrates that F-actin and Arp3 colocalize in space and time. Time in min is on the x-axis and distance in µm is on the y-axis. (B) Histogram of ventral actin wave velocities measured in U2OS cells expressing F-tractin-GFP (n = 42 ventral F-actin waves). To measure ventral F-actin wave velocities, kymographs were made along the trajectory of ventral F-actin wave propagation and the slope of the kymograph was measured. (C) LEFT: TIRFM image of a U2OS cell expressing F-tractin-tdTomato (red) and α_V_ integrin-EGFP (green). The white asterisk is to aid in orientation of the region. Scale bar = 10 µm. CENTER: Images from a time-lapse series of the region highlighted by a yellow box, time in min shown. The white asterisk is to aid in orientation of the region. Scale bar = 10 µm. RIGHT: Kymograph along the trajectory of ventral F-actin wave propagation (highlighted with a yellow arrow at t = 10 min) demonstrates that F-actin temporally precedes α_V_ integrin in ventral F-actin waves. Time in min is on the x-axis and distance in µm is on the y-axis (D) Normalized average intensity of F-tractin-tdTomato (red) and α_V_ integrin-EGFP (green) over time. The average intensity in the green and red channels was measured in the region highlighted by a magenta circle in (C, left) and the values were normalized to the maximal intensity in the time series. To quantify assembly dynamics, we measured the lag time between when ventral F-actin and integrin waves reached half-maximal intensity. To quantify disassembly dynamics, we measured the lag time between when ventral F-actin and integrin waves fell from peak to half-maximal intensity. Lag times are labeled in D. (E) Comparison of lag times with different fluorescent tags. Cells were co-transfected with either F-tractin-tdTomato and α_V_ integrin-EGFP or F-tractin-GFP and α_V_ integrin-tagRFP. Graphs represent lag times between when ventral F-actin and integrin waves reach half-maximal intensity (“Rise to half-max”) as well as when they decrease from peak to half-maximal intensity (“Fall to half-max”). Changing the fluorescent tags did not significantly change the lag time measurements. Values are represented as mean ± SD, n = number of ventral waves analyzed. P-values determined by Student's t-test. (F) Effects of pharmacological perturbation on integrin waves (Bleb = blebbistatin; LatA = latrunculin A; CytoD = cytochalasin D; Rac Inh = NSC23766; PI3K Inh = LY 294002; Dyn = Dynasore hydrate; concentrations below). Cells expressing either α_V_ integrin-tagRFP or α_V_ integrin-EGFP were imaged by TIRFM during perfusion of drugs. We measured the number of waves per min (“frequency”) before and after the drugs were added. We determined the effects of the drugs on integrin waves by dividing the post-drug frequency by the pre-drug frequency for each cell imaged. A value greater than one reflects an increase in wave frequency after drug addition, while a value less than one reflects a decrease in wave frequency after drug addition. n = number of cells analyzed. Data are represented as mean ± SD; P-values determined with Student's t-test.

Since Arp2/3-mediated actin polymerization is coupled to integrin adhesion in lamellipodia, we sought to determine if ventral F-actin waves were also coupled to integrin adhesion. We utilized U2OS cells, a human osteosarcoma cell line, for our studies. When transfected with the F-actin-binding probe F-tractin-tdTomato (Inositol 1,4,5-Trisphosphate 3-Kinase A N66 actin binding domain fused to tdTomato [13]), plated on 5 µg/mL fibronectin, and imaged by Total Internal Reflection Fluorescence Microscopy (TIRFM), 60% of U2OS cells exhibited spontaneous and constitutive moving spots and propagating waves of F-actin at their ventral surface independent of the cell edge. For this study, we defined U2OS “ventral F-actin waves” as transient moving F-actin features localized independent of cell edge that undergo >30% increase in F-tractin average fluorescent intensity, have a lifetime >1 min, an area >1.5 µm^2^. Kymograph analysis showed that U2OS ventral F-actin waves had a mean velocity of 1.61±1.06 µm/min (Figure 1B), comparable to the ventral F-actin wave propagation speeds reported for Dictyostelium and mammalian cells [6], [9], [11], [14]. In addition, co-transfection of F-tractin-tdTomato and Arp3-GFP revealed that Arp3 co-localized with F-actin in U2OS ventral F-actin waves (Figure 1A), similar to Arp2/3 localization in previously reported ventral F-actin waves [11]. Thus, U2OS cells serve as a good model for characterization of Arp2/3-mediated ventral F-actin waves.

To test the hypothesis that ventral F-actin waves are coupled to integrin adhesion, we co-expressed F-tractin-tdTomato along with a fluorescent reporter for a fibronectin receptor, α_V_ integrin-EGFP, and untagged β_3_ integrin. Analysis by TIRFM showed that ventral F-actin waves were associated with α_V_ integrin waves (Figure 1C). Dual color F-tractin and α_V_ integrin kymographs revealed that while both waves propagated with a similar velocity and shape, ventral F-actin waves spatially and temporally preceded integrin waves (Figure 1C, right). In addition to TIRFM, ventral F-actin and integrin waves were also visible with both epifluorescence (Figure S1, Movie S2) and spinning disk confocal imaging (data not shown), suggesting that ventral waves represent a localized increase in the concentration of the proteins and are not a proximity artifact of TIRFM imaging. To quantify the dynamics of ventral F-actin and integrin waves, we plotted the normalized (to maximal in the series) average intensity over time in a region through which a wave propagated (Figure 1D). To determine differences in assembly dynamics, we measured the lag time between when F-tractin and integrin reached half-maximal intensity, and to determine differences in disassembly dynamics we measured the lag time between when F-tractin and integrin decreased from peak to half-maximal intensity. This analysis indicated that ventral F-actin waves rise to half-maximal intensity an average of 83±45s before α_V_ integrin waves (n = 18) and that ventral F-actin waves fall from peak to half-maximal intensity an average of 135±46s before α_V_ integrin waves (n = 16). We confirmed that the measured lag times were not due to differences in the fluorescent protein tag by analyzing ventral F-actin and α_V_ integrin waves in cells expressing F-tractin-GFP and α_V_ integrin-tagRFP (Figure 1E, Movie S1). Thus, ventral F-actin waves are associated with α_V_ integrin waves, however F-actin appears in waves prior to α_V_ integrin.

**Figure 2:**
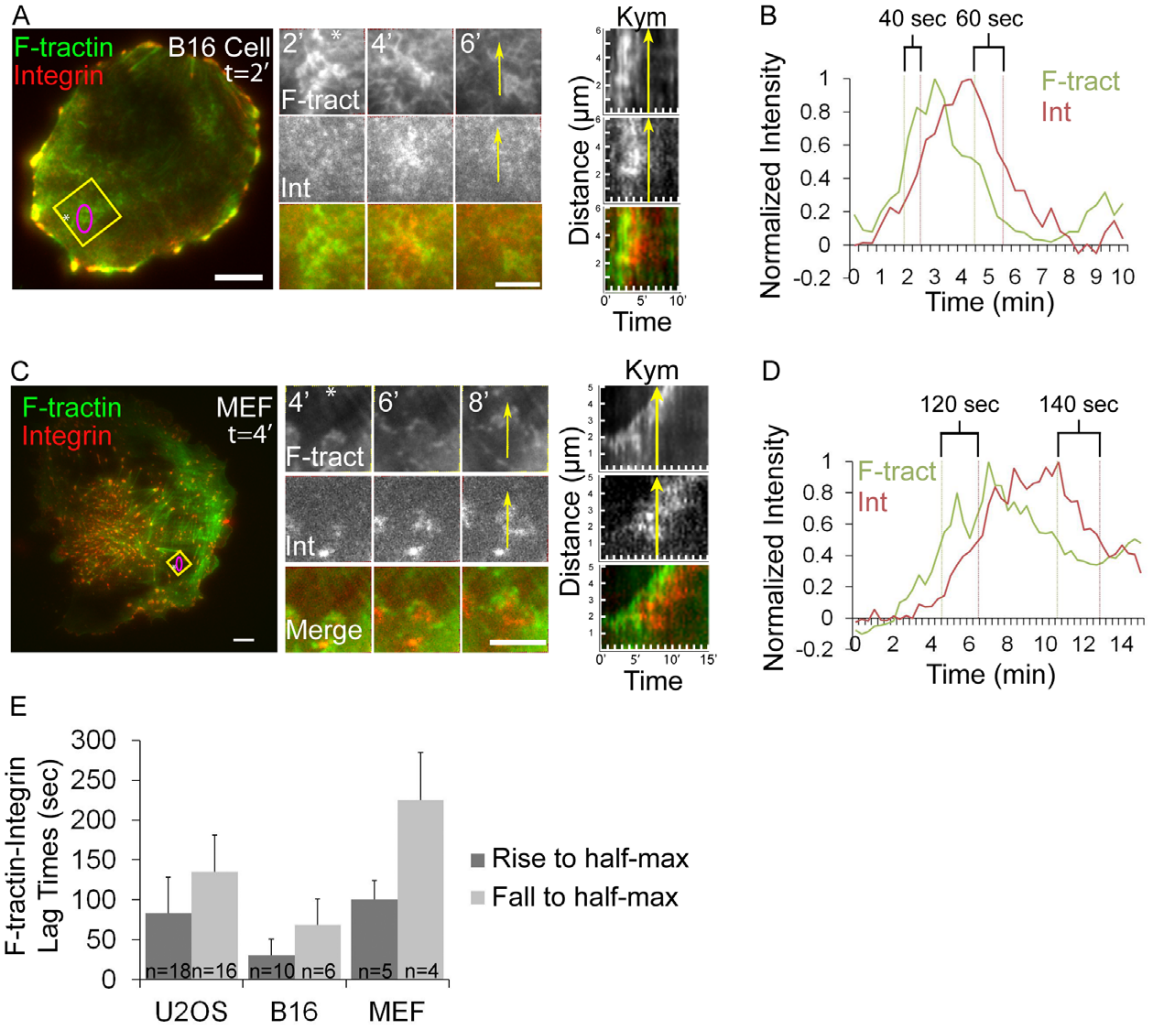
Melanoma and fibroblast cells exhibit ventral F-actin waves followed by integrin waves. (A, C) LEFT: Total internal reflection microscopy (TIRFM) image of a B16-F10 cell (A) or a primary mouse fibroblast (C) co-expressing F-tractin-GFP to label actin filaments (green) and α_V_ integrin-tagRFP (red). The white asterisk is to aid in orientation of the region. Scale bar = 10 µm. CENTER: Images from a time-lapse series of the region highlighted by a yellow box, time in min shown. The white asterisk is to aid in orientation of the region. Scale bar = 5 µm. RIGHT: Kymograph (Kym) along the trajectory of ventral F-actin wave propagation (highlighted with a yellow arrow a t = 6 min(A) and t = 8 min(C)) demonstrates that F-tractin-GFP temporally precedes α_V_ integrin-tagRFP in ventral F-actin waves. (B, D) Normalized average intensity of F-tractin-GFP (green) and α_V_ integrin-tagRFP (red) over time. The average intensity in the green and red channels was measured in the region highlighted by a magenta circle in (A,C, left) and the values were normalized to the maximal intensity in the time series. To quantify assembly dynamics, we measured the lag time between when ventral F-actin and integrin waves reached half-maximal intensity. To quantify disassembly dynamics, we measured the lag time between when ventral F-actin and integrin waves fell from peak to half-maximal intensity. Lag times are labeled in (B, D). (E) Average lag times determined as described in (B,D) for the cell types noted, represented as mean ± SD, n = number of ventral F-actin waves analyzed.

To determine if integrins are associated with ventral F-actin waves in other cell types, we imaged F-tractin-GFP and α_V_ integrin tag-RFP in B16-F10 mouse melanoma cells, which have previously been reported to have ventral F-actin waves [6], as well as primary mouse embryonic fibroblasts (MEFs). Analysis indicated that ventral F-actin waves in B16-F10 cells were associated with integrin waves, although the lag-times between F-actin and α_V_ integrin assembly and disassembly in waves were significantly shorter than those measured in U2OS cells (Figure 2 A–B, E). Primary mouse embryonic fibroblasts (MEFs) infrequently exhibited ventral F-actin waves, although when they were present, they were associated with α_V_ integin waves and exhibited similar assembly/disassembly kinetics as those of U2OS cells (Figure 2 C–E). We conclude that ventral F-actin waves are followed by integrin waves in mammalian cells.

Previous studies have shown that ventral F-actin waves require actin polymerization and PI3K activity, but do not require myosin II activity, and that active Rac1 localizes to ventral F-actin waves [5], [7], [11], [12], [14]. To test the hypothesis that integrin waves are downstream of ventral F-actin waves, we examined the requirement of these activities for U2OS integrin wave formation. We imaged α_V_ integrin-tagRFP or α_V_ integrin-EGFP during perfusion of Latrunculin A (to sequester actin monomers, 2 µM), Cytochalasin D (to cap barbed actin filament ends, 2 µM), LY294002 (to inhibit PI3K, 40 µM), NSC23766 (to inhibit Rac1, 100 µM), or blebbistatin (to inhibit myosin II ATPase, 20 µM). We measured the number of waves per min (“frequency”) and then determined the effects of drugs on integrin waves by normalizing the post-drug frequency to the pre-drug frequency for each cell imaged. Latrunculin, Cytochalasin, NSC23766 and LY294002 all inhibited integrin wave frequency (Figure 1F). In contrast, blebbistatin did not inhibit, but rather enhanced integrin wave frequency, possibly due to Rac activation downstream of myosin II inhibition [15], [16]. To determine if U2OS integrin waves required endocytic recycling of integrins, we imaged α_V_ integrin-tagRFP during perfusion of Dynasore hydrate (to inhibit dynamin GTPase activity, 80 µM). Dynasore hydrate did not affect integrin wave frequency, suggesting that waves do not propagate by dynamin-mediated endocytosis. Together, these results show that Arp2/3-containing ventral F-actin waves are followed by integrin waves in several mammalian cell types, and that, similar to ventral F-actin waves, U2OS integrin waves require actin polymerization, PI3K activity and Rac1 activity, but not myosin II contractility or endocytic recycling, suggesting that ventral F-actin waves and integrin waves are coupled processes.

### U2OS integrin waves are distinct from previously characterized integrin-containing structures

**Figure 3:**
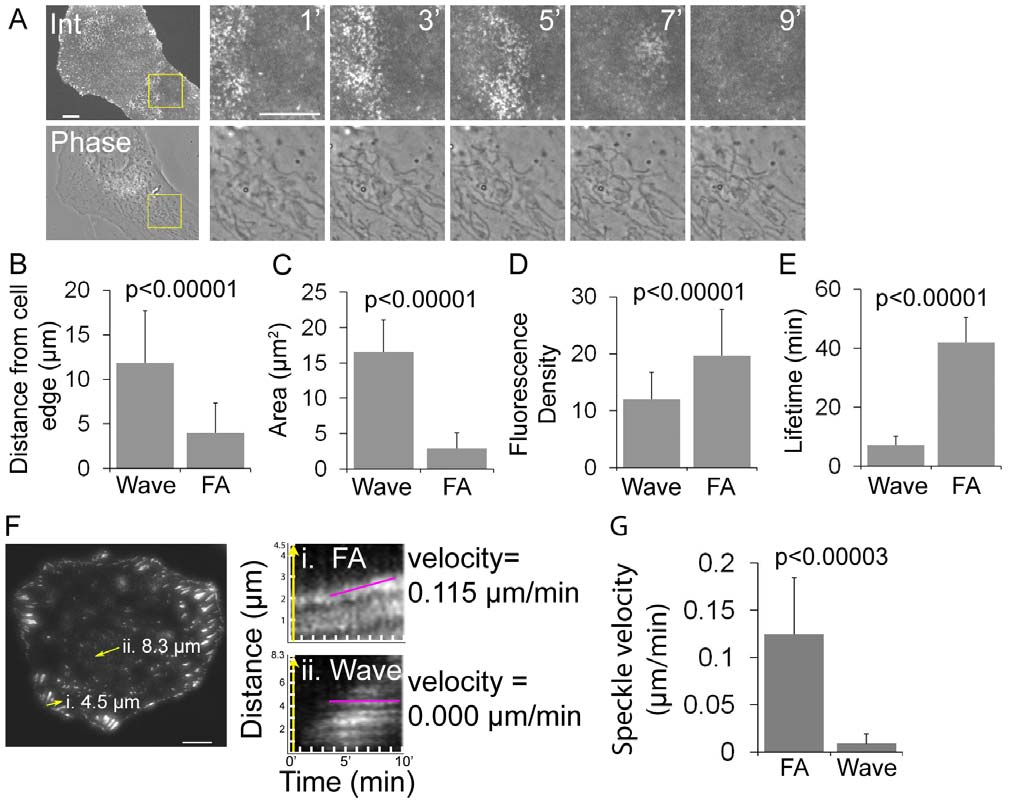
Integrin waves are distinct from podosomes and focal adhesions in U2OS cells. (A) LEFT: Spinning disk confocal and phase-contrast images of a U2OS cell expressing α_V_ integrin-EGFP. Scale bar = 10 µm. RIGHT: Images from a time-lapse series of the region highlighted by a yellow box, time in min shown. The phase contrast image does not exhibit podosome structures as the integrin wave propagates across the ventral surface of the cell. Scale bar = 5 µm. (B) Distance from the cell edge of integrin waves (wave) and focal adhesions (FA) measured in U2OS cells expressing α_V_ integrin-EGFP. Values are represented as mean ± SD; P-values determined with Student's t-test. (C) Area of waves and FAs measured in U2OS cells expressing α_V_ integrin-EGFP. Values are represented as mean ± SD; P-values determined with Student's t-test. (D) Fluorescence density of waves and FAs measured in U2OS cells expressing α_V_ integrin-EGFP. Values are represented as mean ± SD; P-values determined with Student's t-test. (E) Lifetimes of waves and FAs measured in U2OS cells expressing α_V_ integrin-EGFP. FA lifetimes longer than 45 min were recorded as 45 min. Values are represented as mean ± SD; P-values determined with Student's t-test. (F) LEFT: TIRFM image of a cell expressing α_V_ integrin-EGFP. Scale bar = 10 µm. RIGHT: Fluorescent speckle microscopy kymographs of the regions (highlighted with arrows at left) of i) a sliding focal adhesion (FA) and ii) a propagating integrin wave (wave). Magenta lines highlight the path of integrin spekcles within kymographs. Velocity was measured from the slope of the line. (G) Average velocity of integrin speckles within FA or wave structures. Velocity of integrin speckles in FA or waves were measured from the slope of kymographs, as in (F). Integrin speckles in waves remain stationary relative to the substrate. Values are represented as mean ± SD.

We sought to determine if integrin waves were related to previously characterized integrin-containing structures including podosomes, invadopodia and FAs [17]–[19]. Podosomes are peripheral adhesive structures with a core of F-actin surrounded by a small (∼0.5–2 µm) phase-dense ring of FA proteins [17]–[19]. Phase contrast and spinning-disk confocal imaging of α_V_ integrin-EGFP revealed that integrin waves were not phase-dense (Figure 3A), did not exhibit ring-like podosome structure, always exhibited propagating movement, and no podosome-like structures were ever observed in U2OS cells (Figure 1C and Movie S1). Invadopodia are punctate, adhesive structures specialized for ECM degradation that are small (∼0.50 µm), stable (>30 min lifetime), form in the cell center, and contain Arp2/3 and integrins [19]. Although Arp2/3-containing puncta were sometimes observed in the center of U2OS cells, these structures did not directly co-localize with integrins, and were short-lived and dynamic (Movie S3). Thus, in U2OS cells, integrin waves are not related to podosomes or invadopodia.

To determine if integrin waves were similar to or precursors of FAs, we performed TIRFM imaging of α_V_ integrin-EGFP and compared the morphometry and dynamics of integrin waves and FAs. Morphometric analysis of the distance of integrin-containing FAs and integrin waves from the cell edge revealed that, on average, integrin waves were significantly further from the cell edge than FAs (Figure 3B). Integrin waves were also larger, with an average area of 16.6±4.5 µm^2^ as compared with FAs at 2.9±2.3 µm^2^ (Figure 3C). Analysis of fluorescence intensity per unit area showed that α_V_ integrin-EGFP in integrin waves was significantly less dense than in FAs (Figure 3D), suggesting that integrins in integrin waves are less clustered than in FAs. Analysis of time-lapse images revealed that integrin waves were transient structures with an average lifetime of 6.9±3.2 min (n = 26) (Figure 3E) and were never observed to transition into more stable, long-lived structures. In contrast, FAs had an average lifetime of 41.9±8.5 min under our culture conditions. In addition, integrin waves formed and propagated at random sites throughout the ventral cell surface, while FAs formed exclusively in lamellipodia and matured in the lamella near the cell edge. By analyzing waves and FAs with fluorescent speckle microscopy [20], we were able to track integrin speckles within FAs or wave structures (Figure 3F–G). Kymograph analysis showed that integrin speckles within waves remained stationary relative to the substrate (Figure 3F), similar to actin in ventral F-actin waves, which propagate by actin polymerization and treadmilling [7]. This differed from FAs, in which integrin speckles moved coherently relative to the substrate as FAs released from the ECM and slid (Figure 3F and [21]). Furthermore, integrin speckles moved significantly faster in FAs as compared to in waves (Figure 3G). Together, these results support the notion that U2OS integrin waves are distinct from podosomes, invadopodia and FAs.

### Ventral F-actin and integrin waves are ECM-dependent and require a cycle of integrin engagement to and disengagement from the ECM

**Figure 4:**
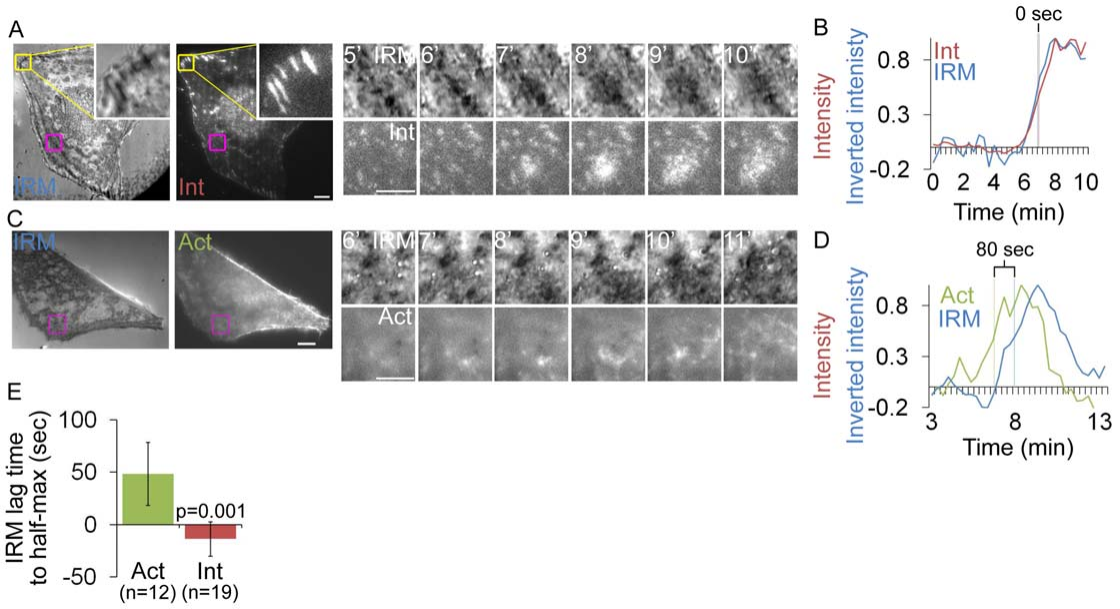
Integrin waves and ventral F-actin waves are visible by Interference Reflection Microscopy. (A) LEFT: Interference reflection microscopy (IRM) and total internal reflection (TIRF) images of a U2OS cell expressing α_V_ integrin-mCherry. Inset (yellow box) shows focal adhesion morphology in IRM and TIRF. Scale bar = 10 µm. RIGHT: Images from a time-lapse series from the regions highlighted by a magenta box, time in min shown. Scale bar = 5 µm. (B) Normalized average intensity of α_V_ integrin-mCherry (red) and inverted IRM intensity (blue) over time in the regions highlighted by a magenta in (A, left). (C) LEFT: Interference reflection microscopy (IRM) and total internal reflection (TIRF) images of a U2OS cell expressing F-tractin-GFP to label actin filaments. Scale bar = 10 µm. RIGHT: Images from a time-lapse series from the regions highlighted by a magenta box, time in min shown. Scale bar = 5 µm. (D) Normalized average intensity of F-tractin-GFP (green) and inverted IRM intesnsity (blue) over time from the region highlighted in (C, left). (E) IRM lag time to rise to half-maximal inverted intensity, relative to rise to half maximal intensity of F-tractin and Integrin. The lag time between when α_v_ integrin or F-tractin reached half-maximal intensity and when IRM reached half-maximal intensity was measured in multiple cells. Actin intensity increase precedes IRM inverted intensity increase, while integrin intensity and IRM inverted intensity increase simultaneously. Values are represented as mean ± SD.

Next, we sought to determine if integrins in ventral waves were interacting with the ECM. To determine if integrin appearance in ventral waves corresponded to the membrane contacting the ECM, we performed Interference Reflection Microscopy (IRM) and TIRFM imaging of cells expressing α_V_ integrin-mCherry. Dark areas in IRM images indicate regions of the plasma membrane in very close (∼10–30 nm) proximity to the coverslip due to destructive interference of reflecting light at areas of cell-substrate contact [22]. TIRF-IRM imaging revealed that the appearance of integrin waves corresponded spatially and temporally with propagating regions of IRM intensity decrease, suggesting that the membrane was coming into closer contact with the substrate (Figure 4A,B; IRM waves are most evident in Movie S4). We inverted IRM images for quantitative analysis and plotted the normalized (to maximal in the series) average intensity over time in a region through which a wave propagated. α_v_ integrin reached half-maximal intensity 13.7±16.4s after IRM (n = 19; Figure 4E). These dynamics did not significantly differ from the dynamics of integrin-tagRFP relative to integrin-EGFP (integrin-tagRFP reached half-maximal intensity 11.1±13.0s after integrin-EGFP; n = 36; p = 0.279). To determine if ventral F-actin waves were coupled to adhesion in cells without expression of exogenous integrin, we performed IRM and TIRFM of cells expressing F-tractin GFP (Figure 4C,D and Movie S4). This revealed that ventral F-actin waves were followed by IRM intensity decrease. Indeed, in plots of F-tractin intensity and IRM inverted intensity over time, F-tractin reached half-maximal intensity 48±30s before IRM (n = 12; Figure 4E). These results suggest that when integrins appear after ventral F-actin waves, they bring the membrane into close proximity to the substrate, and that waves are not an artifact of α_V_β_3_ integrin expression.

**Figure 5:**
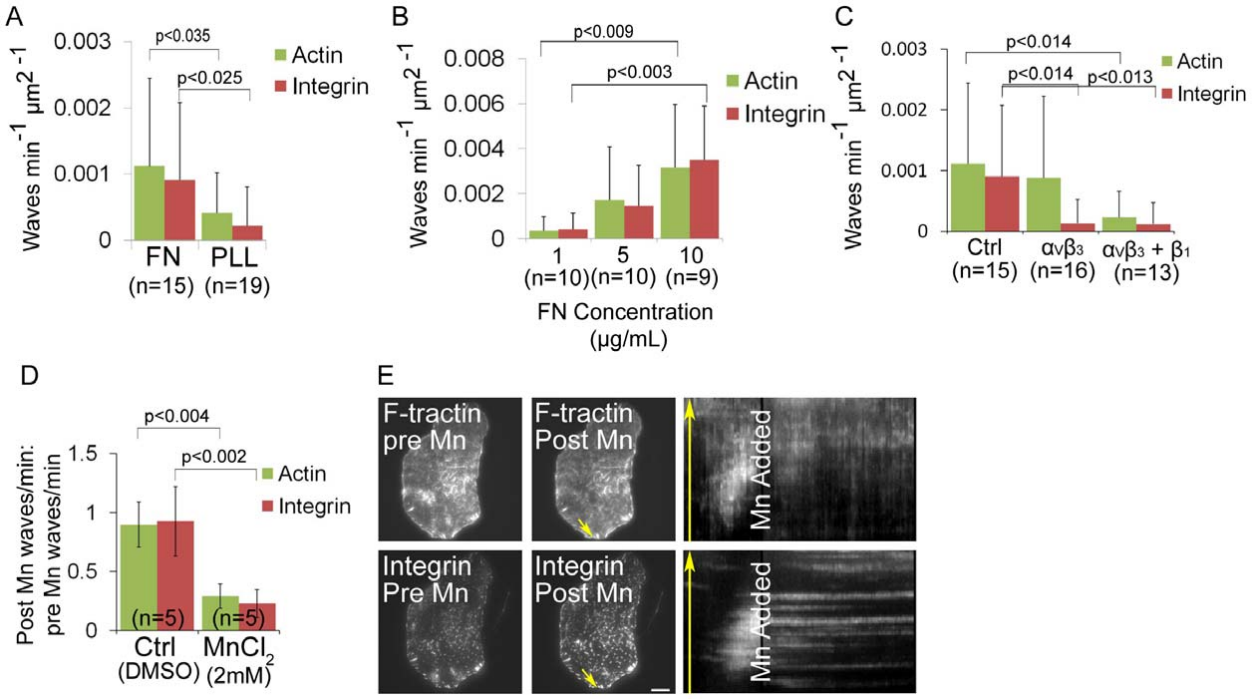
Ventral F-actin and integrin waves require integrin engagement to extracellular matrix (ECM). (A) Ventral F-actin and integrin waves require ECM. U2OS cells expressing F-tractin-GFP to label actin filaments and α_V_ integrin-tagRFP were plated on either 5 µg/mL fibronectin (FN) or 0.01% poly-L-lysine (PLL). In A, B, and C, cells were imaged for 10 min and the average number of waves per min per µm^2^ was measured. P-values determined with Student's t-test. n = number of cells analyzed. (B) Ventral F-actin and integrin waves are sensitive to FN concentration. U2OS cells expressing F-tractin-GFP and α_V_ integrin-tagRFP were plated on increasing concentrations of FN (1 µg/mL, 5 µg/mL and 10 µg/mL). (C) Integrin waves require integrin engagement to ECM. U2OS cells expressing F-tractin-GFP and α_V_ integrin-tagRFP were plated on 5 µg/mL FN in the presence of 20 µg/ml LM609 antibody to block α_V_β_3_ binding to FN (“α_V_β_3_”) or 20 µg/ml LM609 antibody + P4C10 (1∶20 dilution) to block α_V_β_3_ and β_1_ binding to FN (“α_V_β_3_+β_1_”). (D) Effect of MnCl_2_ on ventral F-actin and integrin waves. Cells expressing F-tractin-GFP and α_V_ integrin-tagRFP were imaged 15 min prior to and 30 min after perfusion of 2 mM MnCl_2_. We measured the number of waves per min (“frequency”) before and after MnCl_2_ was added. We determined the effects of MnCl_2_ on waves by dividing the post-drug frequency by the pre-drug frequency for each cell imaged. A value greater than one reflects an increase in wave frequency after drug addition, while a value less than one reflects a decrease in wave frequency after drug addition. n = number of cells analyzed. Data are represented as mean ± SD; P-values determined with Student's t-test. (E) Total internal reflection fluorescence microscopy (TIRFM) images of a U2OS cell expressing F-tractin-GFP and α_V_ integrin-tagRFP immediately prior to (LEFT) and after (CENTER) perfusion of 2 mM MnCl_2_ addition. RIGHT: Kymograph along the trajectory of ventral F-actin wave propagation (highlighted with yellow arrows). MnCl_2_ stops the propagation of both F-actin and integrin waves. Scale bar = 10 µm.

We next sought to determine if ventral F-actin and integrin wave formation and/or propagation require integrin-ECM engagement. To test the requirement of ECM, we plated cells transfected with F-tractin-GFP and α_V_ integrin-tagRFP on either 5 µg/mL fibronectin or 0.01% poly-L-lysine-coated coverslips (Figure 5A). Analysis of ventral F-actin and integrin wave frequency showed that both ventral F-actin and integrin waves were inhibited in cells plated on poly-L-lysine (n = 19) compared with cells on fibronectin (n = 15). Although we imaged cells soon after plating, immunostaining revealed low levels of secreted fibronectin on poly-L-lysine-coated coverslips, suggesting that cells exhibiting ventral F-actin and integrin waves on poly-L-lysine could be responding to secreted ECM (Figure S2A–B). To further test the requirement of ECM for ventral F-actin and integrin waves, we plated cells expressing F-tractin-GFP and α_V_ integrin-tagRFP on increasing concentrations of fibronectin. We found that both ventral F-actin and integrin wave frequency increased in a fibronectin dose-dependent manner (Figure 5B). These results suggest that formation of ventral F-actin and integrin waves is ECM-dependent.

To determine if ventral F-actin and integrin waves require integrin activation, we next plated cells on fibronectin in the presence or absence of the function-blocking LM609 antibody to α_V_β_3_ integrin (Figure 5C) [23]. α_V_ integrin-tagRFP did not localize to paxillin-EGFP labeled adhesions in cells plated on fibronectin in the presence of 20 µg/mL LM609, confirming that 20 µg/mL of LM609 is sufficient to block α_V_β_3_ interaction with fibronectin (Figure S2C). Analysis of ventral F-actin wave frequency showed that integrin waves, but not ventral F-actin waves, were inhibited in cells plated in the presence of 20 µg/mL LM609 (n = 16) compared with control cells (n = 15). To determine if the ventral F-actin waves observed in the presence of LM609 were dependent on β_1_ integrins, we plated cells in the presence of LM609 and the function-blocking P4C10 antibody to β_1_ integrin. This resulted in fewer cells attached to the coverslip, likely due to adhesion defects (data not shown). However, analysis of wave frequency in cells that adhered and spread showed that both ventral F-actin and α_V_ integrin waves were inhibited in the presence of LM609 and P4C10 (n = 13) compared with control cells (n = 15). Thus, ventral F-actin and integrin waves require integrin activation.

Since integrin waves require integrin engagement to the ECM and appear to propagate by treadmilling engagement to the ECM (Figure 3F), we sought to determine if they also required integrin disengagement from the ECM. Mn^2+^ binds to the extracellular metal ion binding sites of integrin, inducing conformational changes correlated with high-affinity binding to ligand; therefore, MnCl_2_ is commonly used to induce integrin activation [24]. We analyzed ventral F-actin and integrin wave frequency in cells expressing F-tractin-GFP and α_V_ integrin-tagRFP and treated with 2 mM MnCl_2_ to induce integrin activation (Figure 5D). This revealed that MnCl_2_ significantly inhibited both ventral F-actin and integrin waves. Furthermore, imaging cells during perfusion revealed that MnCl_2_ produced an immediate effect on ventral F-actin and integrin waves by ceasing their motion and blocking their disassembly, essentially freezing them in place (Figure 5E). Collectively, these results suggest that ventral F-actin and integrin wave formation and propagation require a cycle of integrin engagement to the ECM and disengagement from the ECM. Furthermore, ventral F-actin waves require integrin activation, since both MnCl_2_ treatment and simultaneously blocking α_V_β_3_ and β_1_ inhibited ventral F-actin waves. Together with our observation that integrin waves spatially and temporally follow ventral F-actin waves and require actin polymerization, these results suggest a positive feedback loop between integrin adhesion and Arp2/3-mediated actin polymerization in ventral F-actin waves.

### Ventral F-actin waves contain focal adhesion proteins that assemble and disassemble in a distinct stepwise order

**Figure 6:**
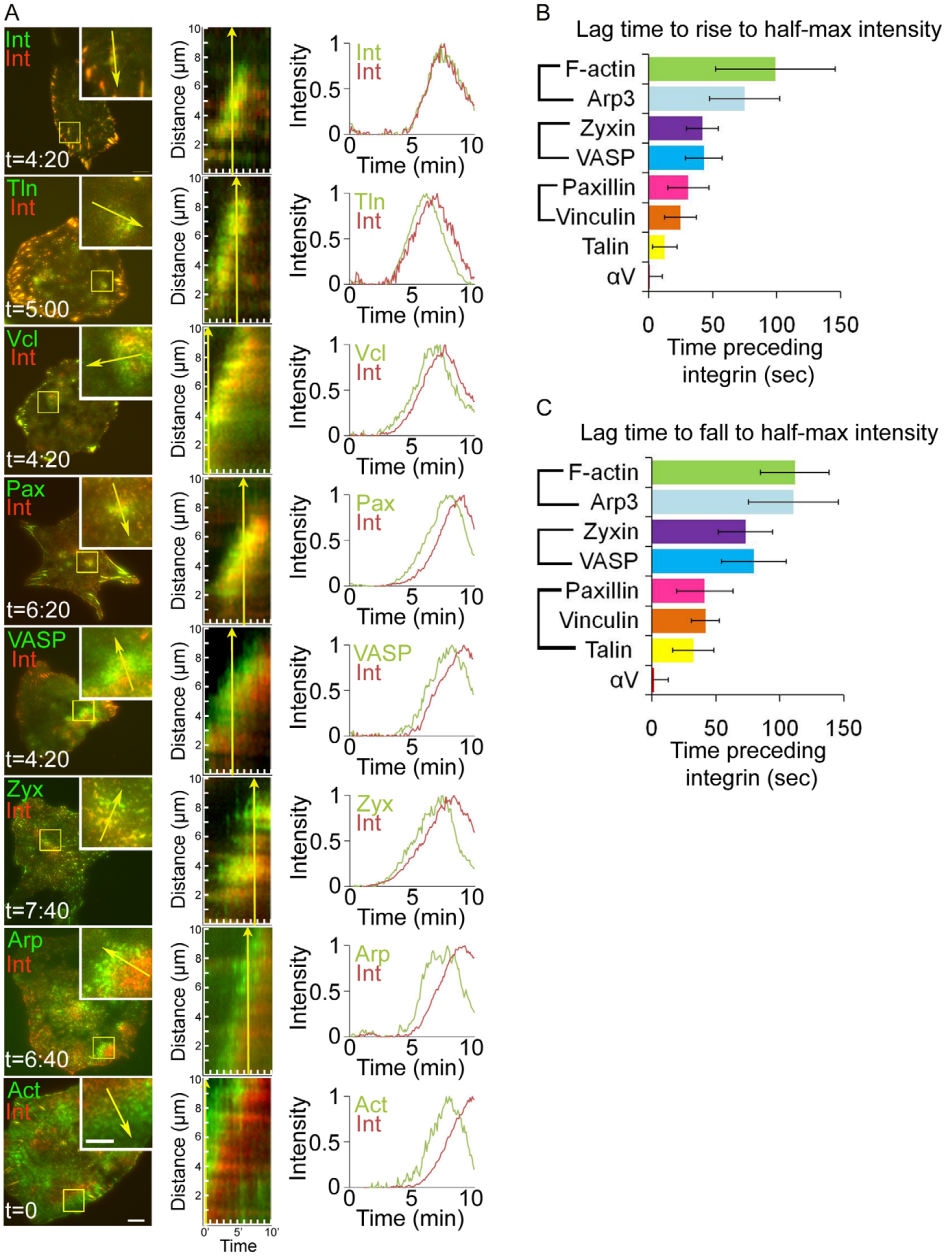
Focal adhesion (FA) proteins assemble into integrin waves in a precise order. (A) α_V_ integrin-tagRFP (Int, red) was co-expressed with the following proteins: α_V_ integrin-EGFP (Int, green), talin-EGFP (Tln, green), vinculin-EGFP (Vcl, green), paxillin-EGFP (Pax, green), VASP-Venus (VASP, green), zyxin-EGFP (Zyx, green), Arp3-GFP (Arp, green) and F-tractin-GFP (Act, green). LEFT: Total internal reflection fluorescence microscopy (TIRFM) images showing an inset (yellow box) of a ventral wave. Still image was taken from a time series at the labeled time. Scale bar = 10 µm. Inset scale bar = 5 µm. Yellow arrow indicates region of kymograph measurement. CENTER: Kymograph along the trajectory of integrin wave propagation. The yellow arrow corresponds to the arrow in (A, left) to demonstrate the direction of wave propagation as well as the time the still image corresponds to in the kymograph. Cells imaged at 20s intervals. Kymograph region = 10 µm. RIGHT: Representative quantification of normalized average fluorescence intensity (“Intensity”) over time for ventral waves in cells imaged at 5s intervals. (B) Average lag time between when fluorescence intensity of FA proteins and α_V_ integrin rise to half-maximal. In B and C, the graphs represent the mean and standard deviation of n>10 integrin wave measurements per condition. Brackets denote lag-times that do not significantly differ from each other as determined by Student's t-test (detailed statistics found in Table S1). (C) Average lag time between when fluorescence intensity of FA proteins and α_V_ integrin fall from peak to half-maximal.

Since actin does not directly bind integrin, we sought to determine what proteins may be involved in coupling actin polymerization to integrin adhesion in ventral F-actin waves. We co-expresssed fluorescently tagged FA adapter and actin-binding proteins together with α_V_ integrin-tagRFP and imaged by TIRFM to identify proteins that localized to integrin waves. This revealed that zyxin-EGFP, VASP-venus, paxillin-EGFP, vinculin-EGFP and talin-EGFP all localized to α_V_ integrin-tagRFP waves as well as to FAs (Figure 6A, left and Figure S4). However, kymograph analysis of time-lapse images revealed that many of these proteins did not temporally coincide with the appearance of α_V_ integrin, but, similar to F-actin, preceded α_V_ integrin appearance in ventral waves (Figure 6A, center). To confirm that FA protein expression did not promote association with ventral F-actin waves, we immunolocalized endogenous paxillin and F-actin in fixed U2OS cells and found that endogenous paxillin was associated with ventral F-actin wave-like structures (Figure S3). However, similar to fluorescently tagged proteins, F-actin and endogenous paxillin did not perfectly overlap; rather, they were separated into a region of F-actin, followed by a region of paxillin and F-actin colocalization, followed by a region of paxillin. Therefore, FA proteins localize to ventral F-actin waves.

To determine the order of protein assembly into ventral F-actin waves, we analyzed the intensity of the various fluorescent FA proteins and α_V_ integrin over time in a region through which a wave propagated (Figure 6A, right), and measured the lag time between when the FA protein and α_V_ integrin intensities rose to half-maximal (Figure 6B). We used co-expression of α_V_ integrin-EGFP with α_V_ integrin-tagRFP as a control. Statistical analysis revealed that proteins were recruited to ventral F-actin waves in five distinct temporal steps: 1. F-actin and Arp3 appear (half maximal intensity reached 99±47 and 75±27s before α_V_ integrin, respectively); 2. Zyxin and VASP appear (half maximal intensity reached 42±12 and 43±14s before α_V_ integrin, respectively); 3. Paxillin and vinculin appear (half maximal intensity reached 31±16 and 25±12s before α_V_ integrin, respectively); 4. Talin appears (half maximal intensity reached 12±10s before α_V_ integrin); 5. Integrin appears (α_V_ integrin-EGFP reached half maximal intensity 1±10s before α_V_ integrin-tagRFP) (Table S1A). Similarly, we determined ventral F-actin wave disassembly order by measuring the lag time between when the FA protein and α_V_ integrin intensities fell from peak to half-maximal (Figure 6C). This showed that wave disassembly was also hierarchical and largely mirrored the assembly process: 1. F-actin and Arp3 dissociate (half maximal intensity reached 112±27 and 110±35s before α_V_ integrin, respectively); 2. Zyxin and VASP dissociate (half maximal intensity reached 73±21 and 80±25s before α_V_ integrin, respectively); 3. Paxillin, vinculin and talin dissociate (half maximal intensity reached 41±22, 42±11 and 32±16s before α_V_ integrin, respectively); 4. Integrin dissociates (half maximal intensity of α_V_ integrin-EGFP reached 2±11s before α_V_ integrin-tagRFP) (Table S1B). Measurements did not differ when images were acquired in the reverse order (i.e. green before red vs. red before green, data not shown). Together, these results show that ventral F-actin waves contain FA proteins that assemble and disassemble in a distinct stepwise order.

## Discussion

**Figure 7:**
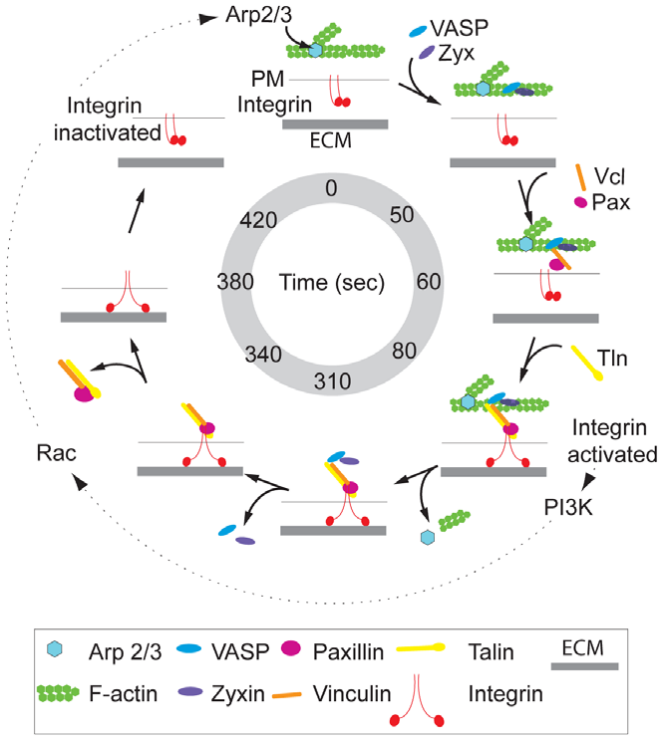
Speculative model of adhesive F-actin wave assembly and disassembly. Speculative model of adhesive F-actin wave assembly and disassembly, based on the average wave lifetime of 7 min. First, Arp2/3 mediates actin polymerization. ∼50s later, the adapter and actin regulatory proteins zyxin and VASP are co-recruited, likely to regulate F-actin barbed end assembly. This is followed rapidly by co-recruitment of the VASP- and actin-binding protein vinculin and its binding partner the adapter protein paxillin at ∼60s. At 80s, the actin and integrin binding protein talin is recruited, possibly by interaction with vinculin. Talin association with ventral F-actin waves then presumably facilitates the inside-out activation of integrin and induces ECM adhesion. ∼310s after initial polymerization in adhesive F-actin waves, F-actin depolymerizes and Arp2/3 dissociates. By 340s, VASP and zyxin co-disassemble, followed by paxillin, vinculin, and talin co-disassembly at 380s. Finally, at 420s integrin dissociates from adhesive F-actin waves by inactivation, and the membrane is no longer tethered to the substrate. A possible positive feedback loop between integrin adhesion and actin polymerization [25] with unknown timing is denoted by dashed arrows, as described in the discussion.

We show here that ventral F-actin waves are associated with integrin-based ECM adhesion in “adhesive F-actin waves.” Adhesive F-actin waves represent a novel type of integrin-mediated adhesion complex that is distinct from previously described integrin-based structures including podosomes, invadopodia and FAs. These findings also support the notion of an inherent coupling between Arp2/3-mediated actin polymerization at the plasma membrane and integrin-based adhesion. We show that integrin waves spatially and temporally follow ventral F-actin waves and require actin polymerization, suggesting that integrin adhesion is downstream of Arp2/3-mediated actin polymerization. However, we find that ventral F-actin waves require a cycle of integrin engagement and disengagement for formation and propagation, since removing fibronectin, blocking α_V_β_3_ and β_1_ integrin activation, or treating with MnCl_2_ inhibited ventral F-actin waves. This suggests that while integrin appearance in waves is downstream of actin polymerization, there could be a positive feedback between integrin engagement and induction of actin polymerization. For example, an integrin-PI3K-Rac signaling cascade has been shown promote Arp2/3 activation and actin polymerization [25] (Figure 7). However, future work will need to address the contribution of downstream targets of integrin signaling to fully understand the relationship between integrin adhesion and actin polymerization in ventral F-actin waves.

Although the function of adhesive F-actin waves is not known, several intriguing possibilities exist. First, adhesive F-actin waves could be a weaker adhesion structure than FAs that could be involved in ameboid migration modes. Alternatively, since they are more dynamic than FAs and sensitive to ECM concentration, adhesive F-actin waves could be a mechanism to sense the surrounding matrix and promote actin polymerization where there is a higher concentration of ECM, thus possibly mediating haptotaxis. Adhesive F-actin waves could also have different roles in different cell types, possibly due to differences in Arp2/3 activators. Regardless of any differences in events upstream of actin polymerization, we have shown that integrin recruitment to ventral F-actin waves is common to the mammalian cells analyzed. These findings support the idea that Arp2/3-mediated actin polymerization at the plasma membrane is inherently coupled to integrin-mediated adhesion, possibly through the molecular interactions of FAK and vinculin or through force-induced changes in integrin conformation by actin polymerization [2], [3], [26].

Our results suggest a speculative hierarchical model of adhesive F-actin wave assembly and disassembly based on the average wave lifespan of 7 min (Figure 7). First, Arp2/3 mediates actin polymerization. ∼50s later, the adapter and actin regulatory proteins zyxin and VASP are co-recruited, likely to regulate F-actin barbed end assembly. This is followed rapidly by co-recruitment of the VASP- and actin-binding protein vinculin and its binding partner, the adapter protein paxillin, at ∼60s. At 80s, the actin and integrin binding protein talin is recruited, possibly by interaction with vinculin. Talin association with ventral F-actin waves then presumably facilitates the inside-out activation of integrin and induces ECM adhesion. ∼310s after initial polymerization in adhesive F-actin waves, F-actin depolymerizes and Arp2/3 dissociates. By 340s, VASP and zyxin co-disassemble, followed by paxillin, vinculin, and talin co-disassembly at 380s. Finally, at 420s integrin dissociates from adhesive F-actin waves by inactivation, and the membrane is no longer tethered to the ECM. The co-assembly and -disassembly of groups of proteins that are known to bind to one another (zyxin and vasp; paxillin and vinculin) suggests they may interact with adhesive F-actin waves as pre-formed complexes, although it is also possible that we are unable to resolve their dynamics at our current image acquisition rate of 5s.

The order of adhesive F-actin wave assembly, in which the recruitment of FA adapter and actin-binding proteins precedes integrin recruitment and activation, appears to differ dramatically from the assembly of FAs. It is well-accepted that integrin activation by talin is the first step in FA assembly, followed by recruitment of cytosolic FA proteins [27]. However, FA assembly and turnover is a complex, hierarchical process involving a series of decision points. Initially, diffraction limited “nascent adhesions” containing β_1_ integrin and paxillin form coupled to lamellipodial actin assembly and independently of myosin II activity, reminiscent of the coupling of actin assembly to integrin adhesion in adhesive F-actin waves. However, nascent adhesions subsequently either disassemble rapidly ∼75s after formation, or undergo myosin II-dependent “adhesion maturation” [1]. Adhesion maturation is characterized by further recruitment of integrins and additional cytosolic proteins to lengthen nascent adhesions into focal complexes and facilitate additional growth into mature FAs. Mature FAs then either disassemble or continue to grow into fibrillar adhesions that remodel ECM [27].

Both nascent adhesion and adhesive F-actin wave formation require actin polymerization and are independent of myosin II; however, in spite of these similarities, the long lag time (∼1 min) between assembly of FA proteins and activation of integrins in adhesive F-actin waves and the fact that these structures never mature into FAs suggest that adhesive F-actin waves are different from nascent adhesions. Since the rapid assembly rate of nascent adhesions (∼1s vs ∼80s for adhesive F-actin wave assembly) has precluded determination of a temporal hierarchy of protein recruitment, it is not yet possible to directly compare nascent adhesion assembly with the adhesive F-actin wave assembly hierarchy demonstrated in this study. Therefore, we cannot rule out the possibility that adhesive F-actin wave assembly may reflect a more general mechanism of adhesion assembly, only on a different time-scale. Future work should aim to determine which FA proteins are required for adhesive F-actin wave propagation and if these proteins have similar roles in FAs and adhesive F-actin waves. This study presents an initial characterization of the morphology, dynamics, ECM-dependence, and assembly mechanism of adhesive F-actin waves and provides compelling evidence that adhesive F-actin waves represent a novel integrin-mediated adhesion complex distinct from previously characterized integrin-containing adhesion structures.

## Materials and Methods

### Cell culture, Transfection, and Reagents

U2OS cells were obtained from American Type Culture Collection (ATCC, Manassas, VA, USA) and maintained at 37°C in McCoy's 5A medium supplemented with 10% FBS (Gibco Labs) at 5% CO_2_. B16-F10 cells were obtained from ATCC and maintained at 37°C in DMEM (Gibco Labs, Carlsbad, CA, USA) supplemented with 10% FBS at 5% CO_2_. Primary Mouse Embryonic Fibroblasts (MEFs) were isolated as follows. Animals were maintained according to the protocol approved by the NHLBI Animal Care & Use Committee on 12/08/2010. Mice were kept on a C57J/BL6 background. E13.5 embryos were dissected from pregnant females, obtained from timed matings, and decapitated before internal organs were removed. Remaining tissue was cut into pieces and incubated 3×10 min in 0.25 mg/ml Trypsin/EDTA (Life technologies, Carlsbad, CA, USA). Single cells were transferred into DMEM supplemented with 20% FBS after each incubation. Pooled suspensions were passed through 100 µm nylon mesh, and cells were pelletted (5 min, 1200 rpm) and plated. Non-adherent cells were removed after 2 h and cultures were maintained in DMEM supplemented with 20% FBS at 5% CO_2_. MEFs were used for experiments at passage 2–3. Transfections were performed using a Nucleofector (U2OS: solution V, program X-001; B16: solution V, program P-020; MEF: solution MEF1, program T-020; Lonza, Basel, Switzerland). For experiments, cells were plated for 16–20 hrs prior to imaging on 22×22 mm #1.5 coverslips pre-coated with 5 µg/ml fibronectin (2 hr at 37°C; Millipore, Billerica, MA). For determining ECM-dependence, 22×22 mm #1.5 coverslips pre-coated with 0.01% poly-L-Lysine (7 min at 20°C, washed 10 times; Sigma-Aldrich, St. Louis, MO, USA). Imaging was performed in growth media without phenol red and supplemented with 30 units/mL Oxyrase. The following pharmacological inhibitors were used: 2 µM Latrunculin A (Invitrogen, Carlsbad, CA, USA), 2 µM Cytochalasin D (Enzo Life Sciences, Plymouth Meeting, PA, USA), 40 µM LY294002 (Calbiochem, Darmstadt, Germany), 100 µM NSC23766 (EMD Chemicals, Gibbstown, New Jersey, USA), 20 µM blebbistatin (Torronto Research Chemicals, North York, Ontario, Canada), 80 µM Dynasore Hydrate (Sigma-Aldrich, St. Louis, MO, USA), 2 mM MnCl_2_ (Sigma-Aldrich, St. Louis, MO, USA).

The following antibodies were used: LM609 (Millipore, Billerica, MA, USA), P4C10 (Millipore, Billerica, MA, USA), rabbit anti-fibronectin (for immunofluorescence at 1∶400; Sigma-Aldrich, St. Louis, MO, USA), mouse anti-paxillin (for immunofluorescence at 1∶250; BD Transduction Labs, Franklin Lakes, NJ, USA). Alexa 488 phalloidin was obtained from (Invitrogen, Carlsbad, CA, USA). Immunofluorescence staining was performed as described in [28]. The following cDNA expression constructs were used: EGFP conjugates of talin, paxillin and zyxin were described previously [21]; vinculin-EGFP, α_V_ integrin-tagRFP, α_V_ integrin-mCherry, α_V_ integrin-EGFP, and VASP-Venus were provided by Mike Davidson (Florida State University, Tallahassee, FL, USA), β_3_ integrin was provided by Mark Ginsberg (UCSD, San Diego, CA, USA); Arp3-GFP was provided by John Hammer (NHLBI, Bethesda, MD, USA); F-tractin-GFP and F-tractin-tdTomato were provided by Mike Schell (Uniformed Services University of Health Sciences, Bethesda, MD, USA).

### Microscopy

Dual-color time-lapse TIRFM of EGFP and tagRFP or td-Tomato tagged proteins in living cells was performed at 37°C using an Apo TIRF 100×1.49 NA oil immersion objective lens (Nikon Instruments, Melville, NY, USA) on an inverted TE2000E2 microscope system (Nikon Instruments, Melville, NY, USA, [29]) with an evanescent field depth of ∼150 nm. Pairs of EGFP (using 488 nm laser illumination) and tagRFP or tdTomato (using 561 nm laser illumination) images were captured in rapid succession at 20s or 5s intervals using an EMCCD (Cascade II:1024; Photometrics, Tuscon, AZ, USA) operated in the 5 MHz readout mode using EM gain. Confocal images of α_V_ integrin-EGFP and phase contrast images were captured using a Plan Apo 100×1.40NA Ph oil immersion objective lens on an inverted TE2000E2 microscope system (Nikon Instruments, Melville, NY, USA, [29]) equipped with a spinning disk confocal scan head (CSU-X; Yokogawa, Tokyo, Japan). Pairs of EGFP and phase contrast images were captured in rapid succession at 20s intervals using a CCD camera (CoolSNAP HQ2; Photometrics, Tuscon, AZ) operated in the 14-bit mode. Interference reflection microscopy was performed using an Apo TIRF 100×1.49 NA oil immersion objective lens (Nikon Instruments, Melville, NY, USA). Illumination was provided by a mercury arc lamp and a 640/30 bandpass excitation filter (Chroma Technology Corp, Bellows Falls, VT, USA) with a multi-bandpass TIRF dichromatic mirror (Chroma Technology Corp, Bellows Falls, VT, USA) and a DIC analyzer (Chroma Technology Corp, Bellows Falls, VT, USA) in the emission filter wheel to reduce image intensity. Pairs of IRM and either mCherry or GFP images were captured in rapid succession at 20s intervals using an EMCCD camera (Cascade II:1024; Photometrics, Tuscon, AZ) operated in the 5 MHz readout mode using EM gain. Fixed cells were imaged using an Apo TIRF100×1.49 NA plan objective lens (Nikon Instruments, Melville, NY, USA) and an inverted TE2000E2 microscope system (Nikon Instruments, Melville, NY, USA, [29]). Cells were imaged with widefield epi-illumination and with TIRFM as described above. Drug perfusion during high resolution imaging was performed using an RC-30 perfusion chamber (Warner Instruments, Hamden, CT, USA) as described in [28].

### Analysis

All image analyses were performed using Metamorph software (MDS Analytical Technologies, Downingtown, PA, USA). Ventral F-actin waves were defined as moving F-actin features that underwent >30% increase in F-tractin average fluorescent intensity, had a lifetime between 1 min and 15 min, and an area >1.5 µm^2^. Kymographs were created from lines drawn along the path of wave propagation, and the velocity of ventral wave propagation was calculated from the slope of these kymographs. Quantitative analysis of the dynamics of EGFP-tagged proteins and α_V_ integrin-tagRFP in ventral waves was performed as follows. Images were background corrected and the average intensity of the entire cell over the time-lapse was fit to a double-exponential for photobleaching correction. Circular regions of at least 1.6 µm^2^ were drawn around waves in the EGFP channel, and the average fluorescent intensity in both channels was recorded for each point in the time-series. The average of intensities of the 8 frames immediately prior to fluorescence increase was subtracted from all measurements in the series and the values were then normalized to the maximal intensity in the series, so that the normalized intensity ranged from 0–1. To compare assembly and disassembly dynamics of different molecules, lag times were measured from these normalized values. To determine differences in assembly dynamics we measured the lag time between when F-tractin and integrin reached half-maximal intensity, and to determine differences in disassembly dynamics we measured the lag time between when F-tractin and integrin decreased from peak to half-maximal intensity. IRM images were inverted, so that for a 12-bit image, an intensity of 0 became 4095 and an intensity of 4095 became 0. TIRF-IRM quantitative analysis of wave dynamics was performed as for fluorescent protein pairs, except using the values for inverted IRM intensity.

## Supporting Information

Figure S1 **Ventral F-actin and integrin waves are visible with widefield epifluorescence microscopy.** RIGHT: Widefield epifluorescent images of a U2OS cell expressing F-tractin tdTomato to label actin filaments (red) and α_V_ integrin-EGFP (green). Scale bar = 10 µm. LEFT: Images from a time-lapse series of the region highlighted by a yellow box, time in min shown. Scale bar = 10 µm.
(TIF)

Figure S2 **Ventral F-actin and integrin waves require integrin engagement to the extracellular matrix (ECM).** (A) Immunostaining for fibronectin of coverslips coated with 0.01% poly-L-lysine (PLL) and incubated in FBS-containing media for 1 hr (LEFT), coated with 0.01% poly-L-lysine and incubated with U2OS cells for 1 hr (CENTER), or coated with 5 µg/mL fibronectin (FN) and incubated with U2OS cells for 1 hr (RIGHT). Scale bar = 10 µm. (B) Quantification of FN immunostaining of coverslips from (A). n = number of images analyzed. (C) Total internal reflection fluorescence microscopy (TIRFM) images of U2OS cells expressing paxillin-GFP and α_V_ integrin-tagRFP plated on 5 µg/mL FN. Scale bar = 10 µm. TOP: Control cell (Ctrl). Paxillin (Pax) and integrin (Int) localize to focal adhesions. BOTTOM: Cell plated in the presence of 20 µg/mL of the function-blocking anti-α_v_β_3_ integrin antibody LM609 (LM609). α_V_ integrin-tagRFP does not localize to paxillin labeled focal adhesions.
(TIF)

Figure S3 **Endogenous Paxillin and F-actin localize to ventral wave structures.** (A) LEFT: widefield (WF) epifluorescence images of a fixed U2OS cell stained with Alexa 488 phalloidin (green) to visualize F-actin and immunofluorescence localization of paxillin (red). Scale bar = 15 µm. RIGHT: Total internal reflection fluorescence microscopy (TIRFM) images of the region highlighted by a yellow box in (A, Left).
(TIF)

Figure S4 **Fluorescent tagged focal adhesion proteins localize to both ventral waves (** **figure 6A** **) and to focal adhesions.** RIGHT: α_V_ integrin-tagRFP (Int, red) was co-expressed with the following proteins: α_V_ integrin-EGFP ((A) Int, green), talin-EGFP ((B) Tln, green), vinculin-EGFP ((C) Vcl, green), paxillin-EGFP ((D) Pax, green), VASP-Venus ((E) VASP, green), zyxin-EGFP ((F) Zyx, green), Arp3-GFP ((G) Arp, green) and F-tractin-GFP to label actin filaments ((H) Act, green). Scale bar = 10 µm. LEFT: Total internal reflection fluorescence microscopy (TIRFM) images showing an inset (yellow box) of focal adhesions (FA). Scale bar = 10 µm.
(TIF)

Table S1 **Statistical analysis of lag times to rise to half-maximal intensity and fall from peak to half-maximal intensity.** U2OS cells co-expressing α_V_ integrin-tagRFP and either F-tractin-GFP (Act), Arp3-GFP (Arp), zyxin-EGFP (Zyx), VASP-venus (VASP), paxillin-EGFP (Pax), vinculin-EGFP (Vcl), talin-EGFP (Tln) or α_V_ integrin-GFP (Int) and imaged by time-lapse total internal reflection fluorescent microscopy (TIRFM). The background-subtracted, normalized (to maximal) intensities of the fluorescent integrin and focal adhesion (FA) proteins were measured in a region through which an integrin wave propagated. (A) Difference in time for α_V_ integrin-tagRFP and fluorescent FA protein intensities to rise above background to half-maximal intensity (Lag time) were determined (B) Difference in time for α_V_ integrin-tagRFP and fluorescent FA protein intensities to fall from peak to half-maximal (Lag time) were determined. n = number of integrin wave measurements. P-values determined by Student's t-test. Bolded values are not statistically significantly different from one another (p>0.05).
(PDF)

Movie S1 **Time-lapse of a U2OS cell expressing F-tractin-GFP to label actin filaments (green) and α_V_ integrin-tagRFP (red) imaged with Total Internal Reflection Fluorescence Microscopy.** The cell was imaged at 20s intervals. The movie plays at 15 frames/second, elapsed time in min∶sec shown. Scale bars = 10 µm.
(MOV)

Movie S2 **Time-lapse of a U2OS cell expressing F-tractin-tdTomato to label actin filaments (red) and α_V_ integrin-EGFP (green) imaged with Epifluorescence.** This movie corresponds to figure S1. The cell was imaged at 20s intervals. The movie plays at 15 frames/second, elapsed time in min∶sec shown. Scale bars = 10 µm.
(MOV)

Movie S3 **Time-lapse of a U2OS cell expressing Arp3-GFP (green) and α_V_ integrin-tagRFP (red) imaged with Total Internal Reflection Fluorescence Microscopy.** The cell was imaged at 5s intervals. The movie plays at 30 frames/second, elapsed time in min∶sec shown. Scale bar = 10 µm.
(MOV)

Movie S4 TOP: Time-lapse of a region in a U2OS cell expressing α_V_ integrin-mCherry and imaged with Interference Reflection Microscopy (IRM, LEFT) and Total Internal Reflection Fluorescence Microscopy (TIRFM, RIGHT). BOTTOM: Time-lapse of a region in a U2OS cell expressing F-tractin-GFP to label actin filaments and imaged with IRM (LEFT) and TIRFM (RIGHT). The cells were imaged at 20s intervals. The movie plays at 15 frames/second, elapsed time in min∶sec shown. Scale bars = 10 µm.
(MOV)
