# Supplementary figures and images for: Adhesive F-actin Waves: A Novel Integrin-Mediated Adhesion Complex Coupled to Ventral Actin Polymerization
Source: PLoS One. 2011 Nov 1;6(11):e26631. doi: 10.1371/journal.pone.0026631 (PMC3206032; doi:10.1371/journal.pone.0026631)

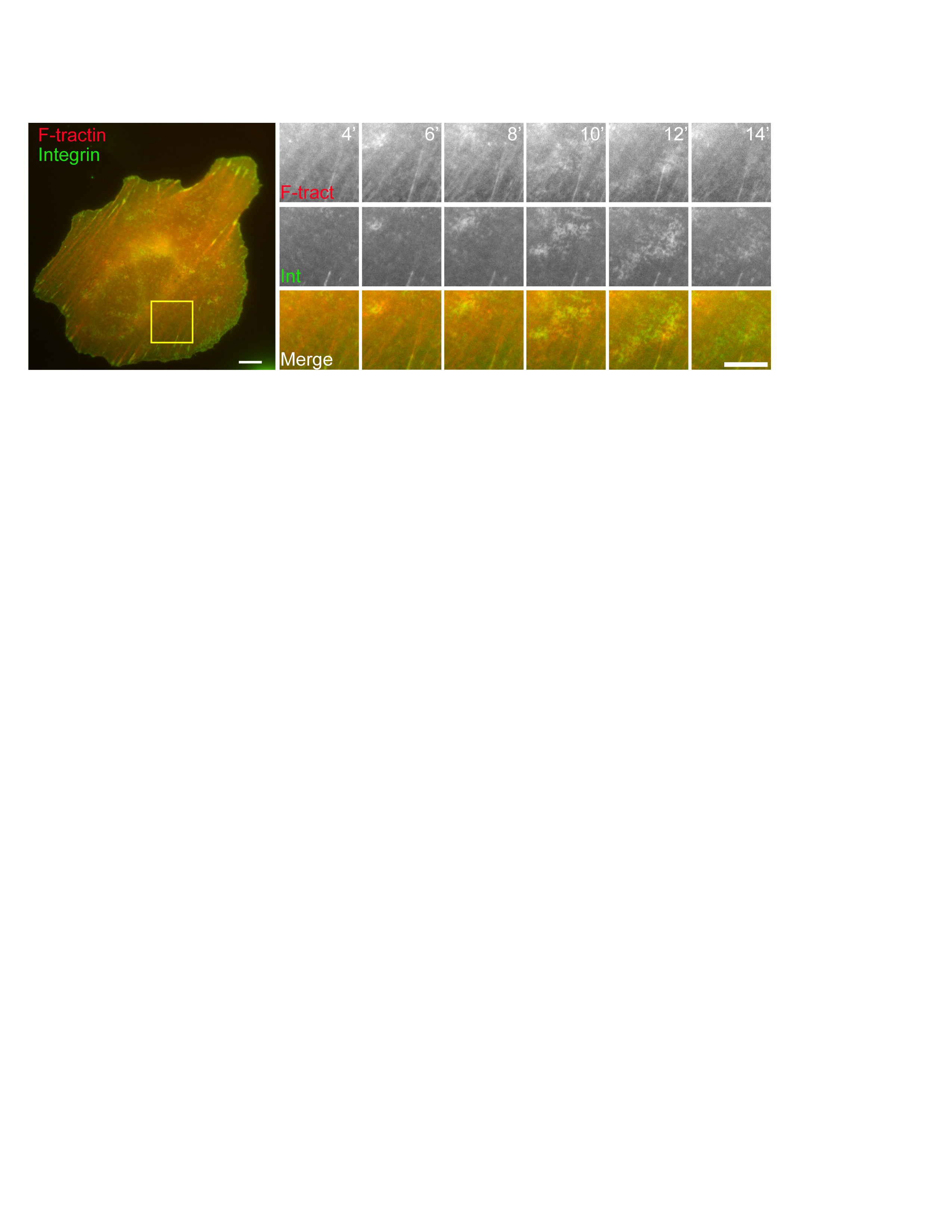

Supplement: Figure S1 — Ventral F-actin and integrin waves are visible with widefield epifluorescence microscopy. RIGHT: Widefield epifluorescent images of a U2OS cell expressing F-tractin tdTomato to label actin filaments (red) and αV integrin-EGFP (green). Scale bar = 10 µm. LEFT: Images from a time-lapse series of the region highlighted by a yellow box, time in min shown. Scale bar = 10 µm. (TIF) [file pone.0026631.s001.tif]

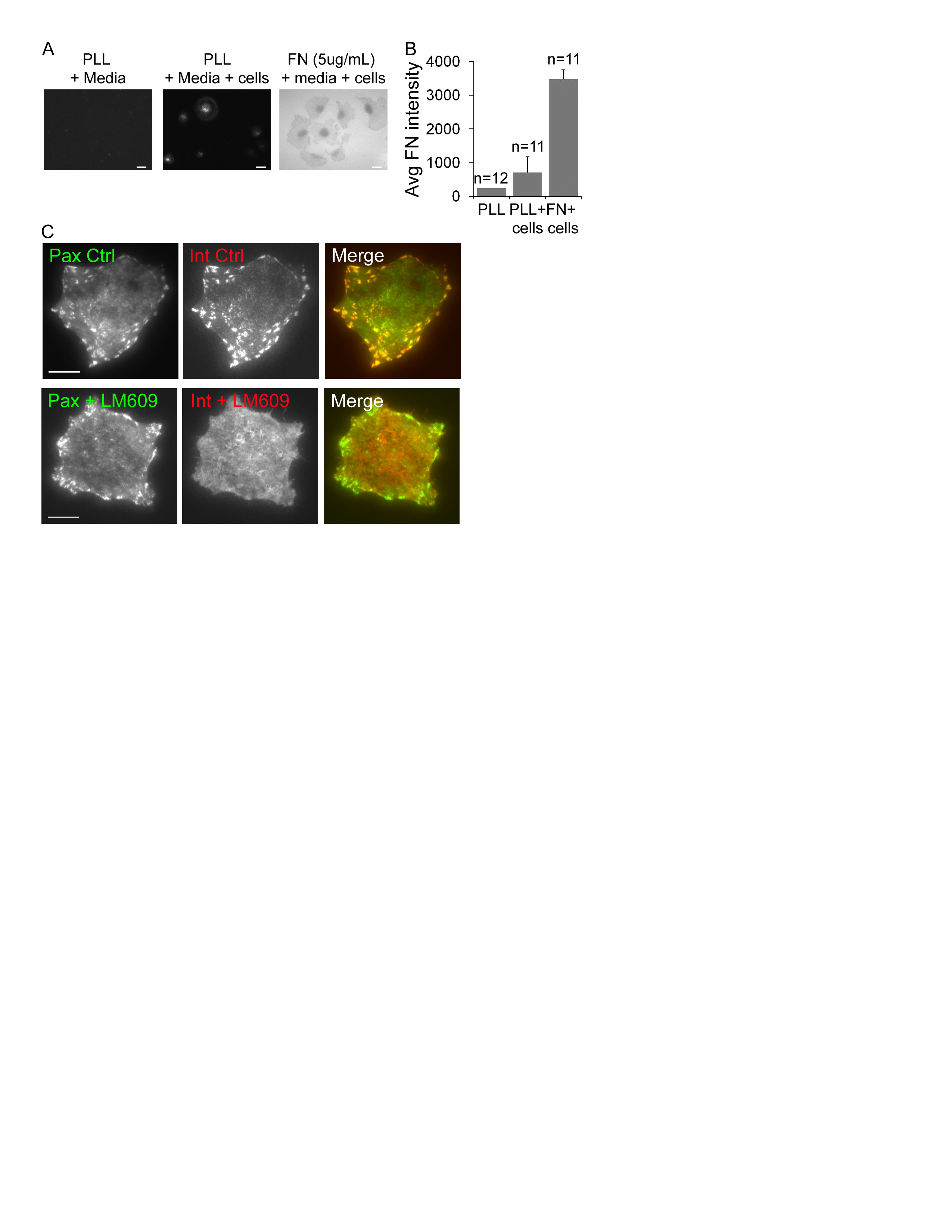

Supplement: Figure S2 — Ventral F-actin and integrin waves require integrin engagement to the extracellular matrix (ECM). (A) Immunostaining for fibronectin of coverslips coated with 0.01% poly-L-lysine (PLL) and incubated in FBS-containing media for 1 hr (LEFT), coated with 0.01% poly-L-lysine and incubated with U2OS cells for 1 hr (CENTER), or coated with 5 µg/mL fibronectin (FN) and incubated with U2OS cells for 1 hr (RIGHT). Scale bar = 10 µm. (B) Quantification of FN immunostaining of coverslips from (A). n = number of images analyzed. (C) Total internal reflection fluorescence microscopy (TIRFM) images of U2OS cells expressing paxillin-GFP and αV integrin-tagRFP plated on 5 µg/mL FN. Scale bar = 10 µm. TOP: Control cell (Ctrl). Paxillin (Pax) and integrin (Int) localize to focal adhesions. BOTTOM: Cell plated in the presence of 20 µg/mL of the function-blocking anti-αvβ3 integrin antibody LM609 (LM609). αV integrin-tagRFP does not localize to paxillin labeled focal adhesions. (TIF) [file pone.0026631.s002.tif]

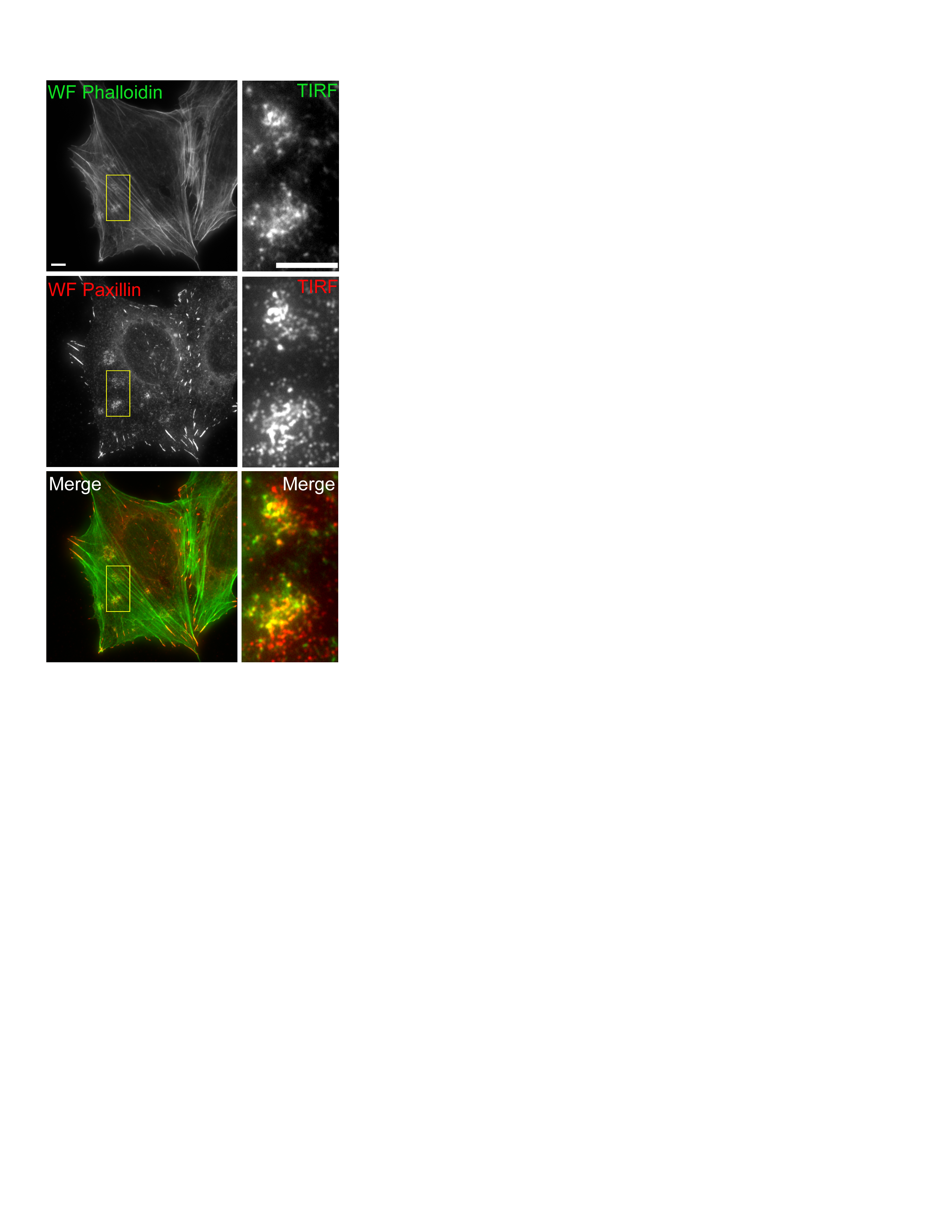

Supplement: Figure S3 — Endogenous Paxillin and F-actin localize to ventral wave structures. (A) LEFT: widefield (WF) epifluorescence images of a fixed U2OS cell stained with Alexa 488 phalloidin (green) to visualize F-actin and immunofluorescence localization of paxillin (red). Scale bar = 15 µm. RIGHT: Total internal reflection fluorescence microscopy (TIRFM) images of the region highlighted by a yellow box in (A, Left). (TIF) [file pone.0026631.s003.tif]

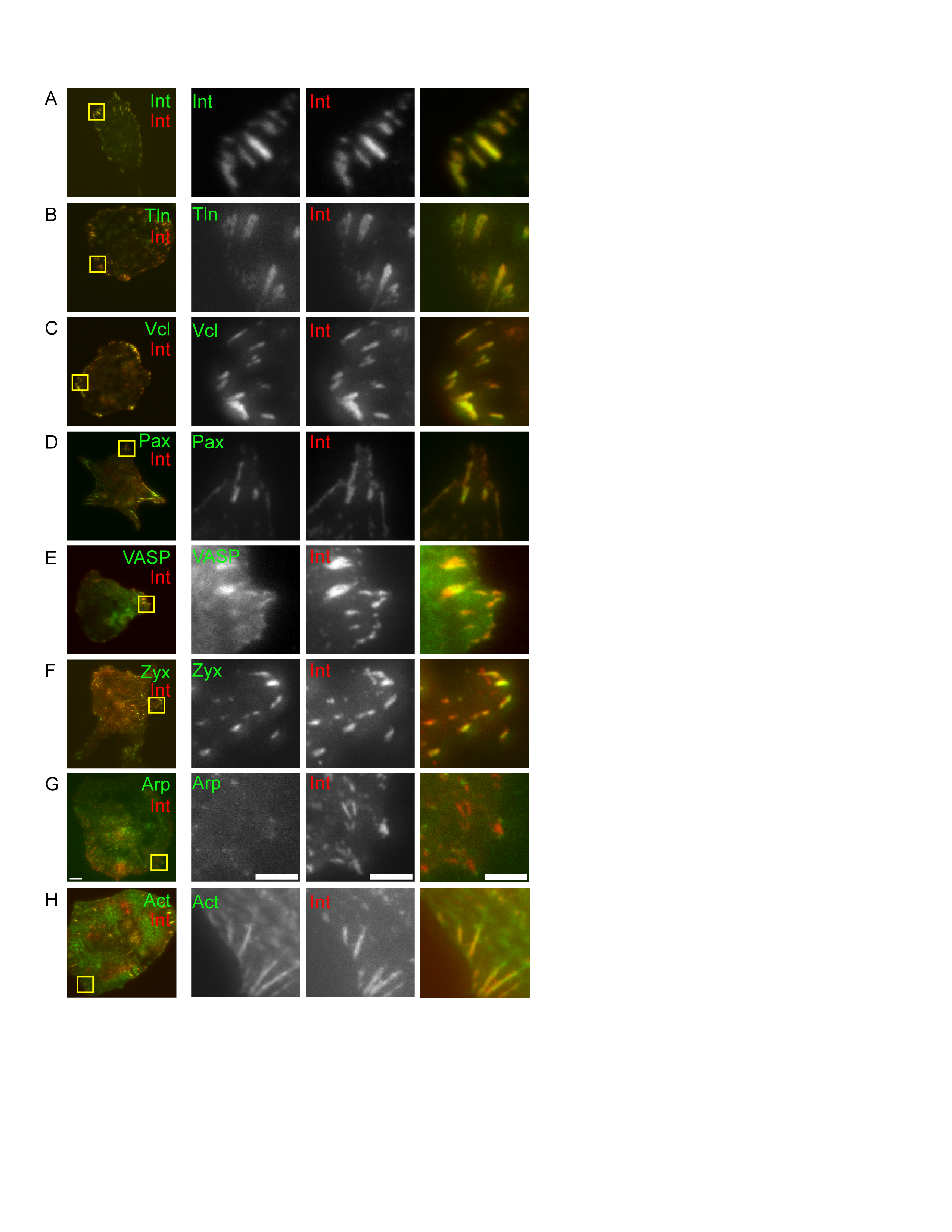

Supplement: Figure S4 — Fluorescent tagged focal adhesion proteins localize to both ventral waves ( figure 6A ) and to focal adhesions. RIGHT: αV integrin-tagRFP (Int, red) was co-expressed with the following proteins: αV integrin-EGFP ((A) Int, green), talin-EGFP ((B) Tln, green), vinculin-EGFP ((C) Vcl, green), paxillin-EGFP ((D) Pax, green), VASP-Venus ((E) VASP, green), zyxin-EGFP ((F) Zyx, green), Arp3-GFP ((G) Arp, green) and F-tractin-GFP to label actin filaments ((H) Act, green). Scale bar = 10 µm. LEFT: Total internal reflection fluorescence microscopy (TIRFM) images showing an inset (yellow box) of focal adhesions (FA). Scale bar = 10 µm. (TIF) [file pone.0026631.s004.tif]
