# Supplementary material for: Adhesive F-actin Waves: A Novel Integrin-Mediated Adhesion Complex Coupled to Ventral Actin Polymerization
Source: PLoS One. 2011 Nov 1;6(11):e26631. doi: 10.1371/journal.pone.0026631 (PMC3206032; doi:10.1371/journal.pone.0026631)
Supplement: Table S1 — Statistical analysis of lag times to rise to half-maximal intensity and fall from peak to half-maximal intensity. U2OS cells co-expressing αV integrin-tagRFP and either F-tractin-GFP (Act), Arp3-GFP (Arp), zyxin-EGFP (Zyx), VASP-venus (VASP), paxillin-EGFP (Pax), vinculin-EGFP (Vcl), talin-EGFP (Tln) or αV integrin-GFP (Int) and imaged by time-lapse total internal reflection fluorescent microscopy (TIRFM). The background-subtracted, normalized (to maximal) intensities of the fluorescent integrin and focal adhesion (FA) proteins were measured in a region through which an integrin wave propagated. (A) Difference in time for αV integrin-tagRFP and fluorescent FA protein intensities to rise above background to half-maximal intensity (Lag time) were determined (B) Difference in time for αV integrin-tagRFP and fluorescent FA protein intensities to fall from peak to half-maximal (Lag time) were determined. n = number of integrin wave measurements. P-values determined by Student's t-test. Bolded values are not statistically significantly different from one another (p>0.05). (PDF) [file pone.0026631.s005.pdf]

A

Molecule	Mean lag time ±SD	P_{Act}	P_{Arp}	P_{Zxy}	P_{VASP}	P_{Pax}	P_{Vcl}	P_{Tln}
Act(n=15)	98.7±46.7							
Arp(n=17)	75.0±27.4	**0.1**						
Zyx(n=21)	41.9±12.5	0.0004	0.0002					
VASP(n=21)	43.1±14.4	0.0004	0.0002	**0.776**				
Pax(n=19)	30.8±16.1	<0.0001	<0.0001	0.021	0.016			
Vcl(n=19)	24.7±12.4	<0.0001	<0.0001	<0.0001	0.001	**0.203**		
Tln(n=25)	12.4±9.70	<0.0001	<0.0001	<0.0001	<0.0001	<0.0001	0.001	
Int(n=18)	0.83±9.90	<0.0001	<0.0001	<0.0001	<0.0001	<0.0001	<0.0001	0.0005
One Way ANOVA	P_{all8}<0.0001							

B

Molecule	Mean lag time±SD	P_{Act}	P_{Arp}	P_{Zxy}	P_{VASP}	P_{Pax}	P_{Vcl}	P_{Tln}
Act(n=12)	111.7±26.8							
Arp(n=14)	110.4±35.3	**0.915**						
Zyx(n=20)	73.0±21.1	0.0004	0.002					
VASP(n=12)	79.6±25.2	0.0006	0.017	**0.456**				
Pax(n=13)	41.2±22.2	<0.0001	<0.0001	0.0004	0.0006			
Vcl(n=12)	41.7±10.9	<0.0001	<0.0001	<0.0001	0.0002	**0.942**		
Tln(n=15)	32.3±16.0	<0.0001	<0.0001	<0.0001	<0.0001	**0.247**	**0.09**	
Int(n=15)	1.76±10.8	<0.0001	<0.0001	<0.0001	<0.0001	<0.0001	<0.0001	0.0005
One Way ANOVA	P_{all8}<0.0001							
